# Innovative approaches for vaccine trials as a key component of pandemic preparedness – a white paper

**DOI:** 10.1007/s15010-024-02347-1

**Published:** 2024-07-17

**Authors:** Ullrich Bethe, Zoi D. Pana, Christian Drosten, Herman Goossens, Franz König, Arnaud Marchant, Geert Molenberghs, Martin Posch, Pierre Van Damme, Oliver A. Cornely

**Affiliations:** 1 Institute of Translational Research, Cologne Excellence Cluster on Cellular Stress Responses in Aging-Associated Diseases (CECAD), Faculty of Medicine and University Hospital Cologne, University of Cologne, Herderstrasse 52, 50931 Cologne, Germany; 2 Center for Integrated Oncology Aachen Bonn Cologne Düsseldorf (CIO ABCD) and Excellence Center for Medical Mycology (ECMM), Department I of Internal Medicine, Faculty of Medicine, University Hospital Cologne, University of Cologne, Cologne, Germany; 3 Partner Site Bonn-Cologne, German Centre for Infection Research (DZIF), Cologne, Germany; 4 Medical School, European University of Cyprus, Nicosia, Cyprus; 5 Institute of Virology, Charité Universitätsmedizin Berlin, Berlin, Germany; 6 Laboratory of Medical Microbiology, Vaccine & Infectious Disease Institute and Biobank Antwerp, University of Antwerp, Wilrijk, Belgium; 7 Center for Medical Data Science, Institute of Medical Statistics, Medical University of Vienna, Vienna, Austria; 8 European Plotkin Institute for Vaccinology, Université libre de Bruxelles, Brussels, Belgium; 9 Interuniversity Institute for Biostatistics and Statistical Bioinformatics, Data Science Institute, KU Leuven and Hasselt University, Wilrijk, Belgium; 10 Centre for the Evaluation of Vaccination, VACCINOPOLIS, Vaccine and Infectious Disease Institute, University of Antwerp, Wilrijk, Belgium

**Keywords:** Adaptive platform trial, Bayesian, Concurrent controls, Enrichment, Frequentist, Interpandemic interval, Modelling pandemic preparedness, Prevention, Randomized clinical trial, Response-adaptive randomization, Simulation, Stakeholders, Trial design, VACCELERATE, Vaccines

## Abstract

**Background:**

WHO postulates the application of adaptive design features in the global clinical trial ecosystem. However, the adaptive platform trial (APT) methodology has not been widely adopted in clinical research on vaccines.

**Methods:**

The VACCELERATE Consortium organized a two-day workshop to discuss the applicability of APT methodology in vaccine trials under non-pandemic as well as pandemic conditions. Core aspects of the discussions are summarized in this article.

**Results:**

An “ever-warm” APT appears ideally suited to improve efficiency and speed of vaccine research. Continuous learning based on accumulating APT trial data allows for pre-planned adaptations during its course. Given the relative design complexity, alignment of all stakeholders at all stages of an APT is central. Vaccine trial modelling is crucial, both before and in a pandemic emergency. Various inferential paradigms are possible (frequentist, likelihood, or Bayesian). The focus in the interpandemic interval may be on research gaps left by industry trials. For activation in emergency, template Disease X protocols of syndromal design for pathogens yet unknown need to be stockpiled and updated regularly. Governance of a vaccine APT should be fully integrated into supranational pandemic response mechanisms.

**Discussion:**

A broad range of adaptive features can be applied in platform trials on vaccines. Faster knowledge generation comes with increased complexity of trial design. Design complexity should not preclude simple execution at trial sites. Continuously generated evidence represents a return on investment that will garner societal support for sustainable funding. Adaptive design features will naturally find their way into platform trials on vaccines.

## Introduction

In the context of pandemic preparedness, WHO postulates that the methodology of ever-warm adaptive platform trials (APTs) be rigorously applied, both in interpandemic intervals and in crises [1]. WHO also calls for optimal scientific and ethical design, including advanced statistical methods [1].

In the vaccines arena, APT methodology has not been leveraged widely. So far, the adaptiveness of vaccine platform trials is either confined to the optional addition of approved vaccines and new schedules [2], or there is no built-in ability to pivot to pandemic mode [3].

In February 2023, the European Commission, Directorate-General Research and Innovation, voiced the need for a “REMAP-CAP-type of continuous trial for vaccines” [4]. REMAP-CAP is an ever-warm, i.e., continuously enrolling, platform trial with certain adaptive design features. However, it focuses on treatment, not on vaccination [5].

The VACCELERATE Consortium was established in 2021 as an integral component of the European Union’s response to the COVID pandemic. VACCELERATE is a research infrastructure for vaccine trials. Its core activities comprise a Europe-wide network of more than 490 clinical trial sites, a registry of > 100,000 volunteer vaccinees and the conduct of three cross-border non-adaptive vaccine trials [6–10].

On January 18th and 19th 2024, University Hospital Cologne organized the VACCELERATE APT Workshop as an initial step in aligning APT stakeholders [11, 12]. With the aim of determining the full potential of APT methodology for pandemic preparedness, the workshop brought together attendees from a wide range of disciplines and backgrounds to discuss the application of APT methodology in vaccine trials.

Core aspects of workshop discussions are reflected in the present white paper, promoting a paradigm shift from a traditional two-arm randomized clinical trial (RCT) towards a continuously learning adaptive platform trial [13]. Authors describe various options for a vaccine APT in pandemic preparedness, but explicitly not a ready-to-use trial. No claim is made to include all opinions voiced during preparations, at the in-person workshop and during manuscript writing.

## Methods

The two-day invitation-only workshop was organized to ensure early involvement and alignment of all important stakeholders for a vaccine APT that serves the objective of pandemic preparedness [11, 12]. Attendees included statisticians, vaccinologists, vaccine immunologists, virologists, microbiologists, clinical trialists, patient advocates, public health experts, regulators, members of ethics committees, and funders.

Parallel break-out sessions were prepared in small groups prior to the workshop and covered the topics of statistics and methodology for APTs, vaccine immunology, interpandemic research questions, vaccine trial conduct, and the set-up of a consortium focused on vaccine APTs. At the in-person meeting, findings from the break-out sessions were discussed in interactive plenaries. These were complemented by pitch presentations on European instruments in pandemic preparedness, pathogens with the highest potential causing the next pandemic, regulators’ perspective on APTs and FAIR (Findable, Accessible, Interoperable, Reusable) data.

## Results

### Statistics and APT methodology

A platform trial has the potential to speed up the evaluation of medicinal products in general, and vaccines in particular, because of the shared use of infrastructure, e.g., shared clinical trial sites, data management, endpoint elicitation or monitoring. In addition, the standardised framework of a platform trial enables comparisons between investigational arms and increases statistical efficiency by sharing common control data. In the context of vaccine development, in both an interpandemic and pandemic context, platform technology needs to be tailored to the specifics of an infectious disease affecting potentially large populations, against the background of rapid evolution of both the mutating virus and the development of natural and vaccine-induced immunity [14].

An APT framework tailored to the specific context of the development of vaccines for infectious diseases, is ideally suited to improve efficiency and speed of candidate vaccine evaluation [11, 15–17]. There are a few relatively recent examples [3, 18].

A key element of an APT is the continual ability to learn and benefit from incoming data. Thorough planning is needed on which adaptations based on which data are beneficial (Table 1). By means of clinical trials simulation [19], the usefulness of potential adaptations should be explored and evaluated upfront in iterative discussions with important stakeholders such as funders, regulators, healthy populations (typical for vaccine trials), patients, etc. In different phases of the pandemic, different adaptations might be of interest, e.g., in the early phase the main objective might be to identify one safe, immunogenic and efficacious vaccine as quickly as possible. For regulatory acceptance, it is crucial to reach pre-agreement on key design aspects [12], such as definition of the estimand of the primary endpoint, and multiplicity control within and across sub-studies [19].

**Table 1:** Spectrum of adaptations in adaptive platform trials

Adaptation	Description
Adding of trial arms	Arms with new vaccines can be added as new sub-studies.
Response-adaptive randomization	Based on outcome data (“response” as broad term) response-adaptive allocation can be used to increase allocation to more promising vaccines.
Early stopping for efficacy or futility	Adaptive interim analyses may allow to quickly graduate promising vaccines and drop others for futility or safety, thereby maximally protecting participants and optimizing use of resources.
Sample size reassessment	Modifying the sample size based on accumulating trial data. These data may be blinded or unblinded.
Subgroup selection	At adaptive interim analysis more promising subgroups can be selected, e.g., based on endpoints reached early on using a CoP.
Regimen selection	If different regimens (doses, fractionations, routes, schedules (spacing, heterologous), interchangeability) of the same vaccine are investigated, more promising regimens can be selected at interim analysis.

CoP, correlate of protection

An APT builds on the well-known features of “traditional” RCTs, in particular experimentation, randomization, and blinding. In addition, an APT offers the flexibility of adaptation rules and the integration of information from other relevant data sources, such as sentinel surveys and other epidemiological studies that provide information on serology, variants, infection, and other endpoints in general populations. Data from post-marketing studies on both effectiveness and safety can also be integrated. The use of natural history data, including the longitudinal evolution of immunity over time, in an unvaccinated but exposed or challenged population, or in a population vaccinated in previous cycles with earlier generation vaccines will offer a valuable enhancement. Mathematical modelling, combined with expertise in health economics, will support the design, dynamic adaptation, and assessment of vaccination benefits at both an individual and population level [20]. For example, certain age-defined population subgroups may benefit strongly from vaccination at an individual level because of high risks for severe disease but may contribute less to the population benefit because of comparatively limited contact patterns, and vice versa. Such findings can be used to prioritize high-risk subgroups in the trial, potentially leading to accelerated approval, differentially across subgroups. This ties in with the development of rules for responsive adaptive randomisation, developed to favour promising arms as soon as possible, potentially tied to specific subgroups of a population. Overall, this suggests the development of a framework for vaccine trial simulation embedded in population modelling and simulation, which can feed into fast and efficient trial design and adaptation rules.

A vaccine APT should collect a variety of clinical endpoints (Table 2). Even when one of these is considered as primary, several combined endpoints can be used to gauge efficacy. The choice of primary endpoint could shift over time. Furthermore, apart from classical – marginal – vaccine efficacy, it will be relevant to estimate conditional vaccine efficacy (Table 2). This calls for a careful choice of estimands [21, 22]. In addition to clinical endpoints, markers should be collected with the potential to be validated as correlates of protection (Table 2). At the onset of the trial program for a new pathogen, the simultaneously collected clinical endpoints and markers can be used to validate one or more markers as correlate of protection (CoP, for examples see Table 2), after which preliminary conditional marketing approval can be granted, with subsequent revision or confirmation once sufficient clinical endpoint data has been collected. To this end, surrogate endpoint evaluation methodology, [23] specialized to the vaccine case, must be used.

**Table 2:** Relevant terms and statistical concepts

Term / Concept	Description
Platform Trial	A multi-armed clinical trial that allows trial arms to be added or dropped during the course of the trial.
Adaptive Design	“A clinical trial design that offers pre-planned opportunities to use accumulating trial data to modify aspects of an ongoing trial while preserving the validity and integrity of that trial” [37].
Power	Depending on the main objective of the APT, different power definitions can be used to determine the sample sizes needed. E.g., power may be defined as the probability to detect (i) at least one efficacious vaccine or (ii) all vaccines with a certain effect size.
Type 1 Error Control	Pre-agreement is needed on how to address multiplicity due to testing several intervention arms versus control.
Concurrent and non-concurrent control data	Concurrent: control data collected in parallel to an intervention arm. Non-concurrent: control data for a specific intervention of patients who were recruited before that intervention was included in the platform. Even though statistical modelling can be used to incorporate both non-concurrent and concurrent control data, there may be regulatory concerns because of potential time drifts. Therefore, it is advised to have sufficient concurrent control data for all intervention arms [38, 39].
Clinical endpoints for vaccine trials	Infection, symptomatic infection, hospitalization, ICU admission, mechanical ventilation, mortality.
Conditional vaccine efficacy	The protective effect on a later or more serious clinical endpoint given that an earlier or less serious one has occurred. A vaccine with modest efficacy against prevention but strong efficacy against more severe endpoints may be considered successful, given the substantial benefit at the individual and societal level.
Correlates of protection (CoP)	Markers that are considered as surrogate parameters of clinical endpoints, e.g., humoral immunity (e.g., binding antibodies, neutralizing antibodies (in a live virus or pseudovirus assay)), but also cellular immunity (e.g. specific T-cell response in an ELISPOT assay). The APT may be set up to allow validation of markers as CoP. It is important to distinguish between a CoP at individual level, i.e., how well does a CoP predict someone’s clinical endpoint or endpoints, given their vaccination status, and a surrogate at trial level, i.e., how well does the vaccination effect ascertained on a surrogate predicts the corresponding vaccination effect on one or more clinical endpoints.
Clinical Trial Simulation	Based on different scenarios, the operating characteristics of different design options are evaluated by simulating the course of a platform trial ten thousand times, e.g., to tailor the adaptation and decision rules and determine sample sizes and power. Ready-to-go simulation programs to conduct complex clinical trial simulations should be put in place to initiate and adapt the APT when switched to the pandemic mode and new scenarios must be evaluated promptly.
Central Data Environment	Prospectively collected data in an APT should be brought together in a common place for efficient analysis but with appropriate firewalls, e.g., if different sponsors are involved.

ICU, intensive care unit; APT, adaptive platform trial; CoP, correlate of protection; IDMC, independent data and safety monitoring committee

These developments should take place in continual dialogue with regulatory authorities [24–26].

Effectiveness and prevalence may change over time. Considering both, the time from vaccination to infection may change due to waning or new pathogen variants, resulting in non-proportional hazards [27]. Also, circulating variants and risk seeking behaviour may change over time [28]. In APTs running over longer time periods, time trends need to be carefully addressed.

The selection and updating of study arms require a transparent decision-making process. Control arms can be shared between studies, data may be collected concurrently or be of a historic control type [15]. When vaccines are approved or updated, previous intervention arms may become active control arms. Especially in a pandemic setting, with eventually extremely high levels of prior natural infection and/or vaccination, ‘control’ should be defined against such serological background.

**Fig. 1:**
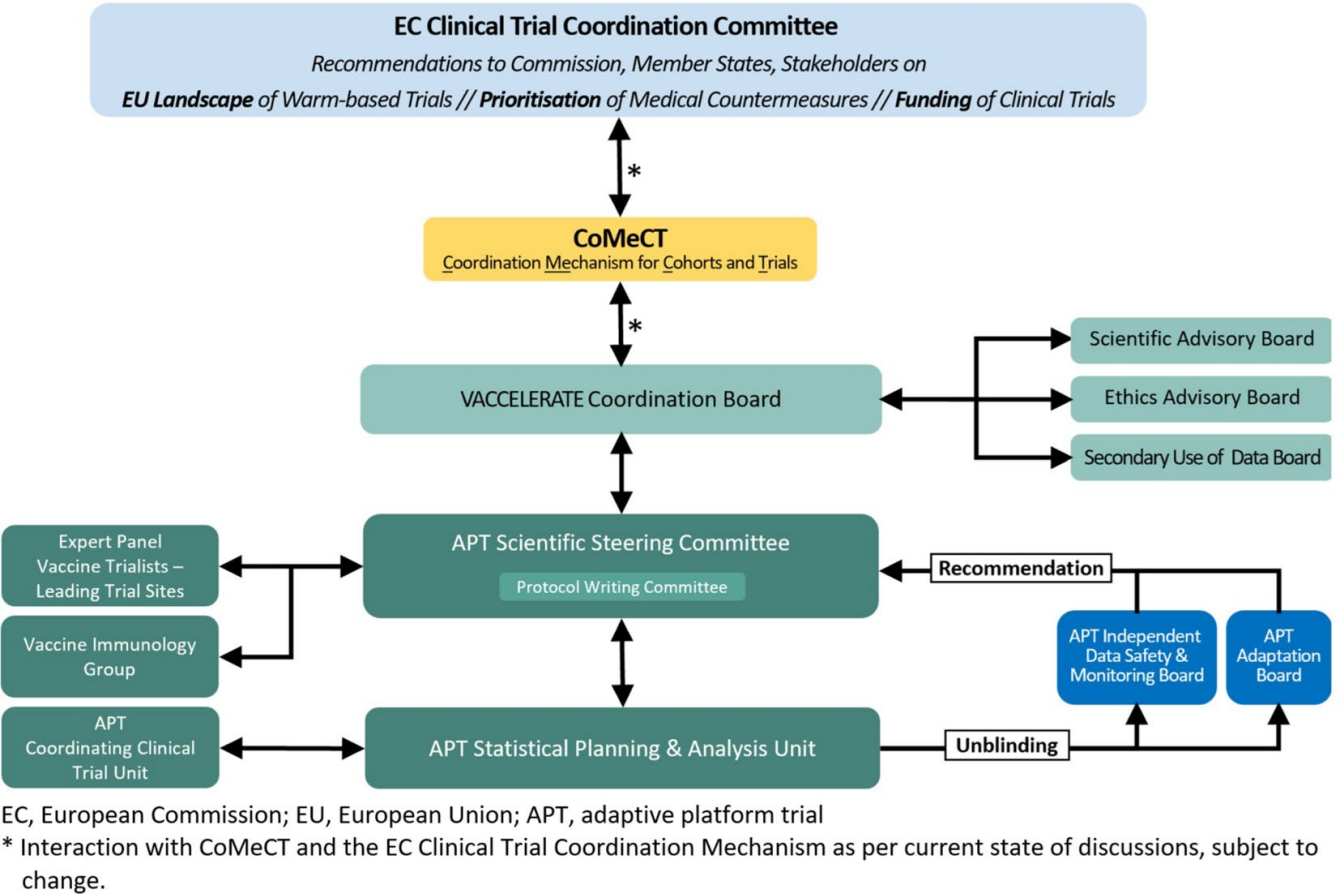
Exemplary setup of a VACCELERATE APT on vaccines

Co-creation between researchers and health authorities, in particular regulators, is essential to ensure a properly functioning infrastructure in both methodological and operational terms. The complexity and flexibility of a vaccine APT require establishing additional dedicated governing bodies [11], with statisticians represented in these, including the Steering Committee, Protocol Writing Committee, IDMCs, Adaptation Board, and a board to regulate the secondary use of data (Fig. 1). The combination of clinical trial and real-world data (RWD) requires statistical expertise of various types in the statistical planning and analysis. Prospectively collected APT data should be brought together in a common data centre for an efficient analysis, with appropriate firewalls if several sponsors are involved. When combining several data sources including RWD, it might not be feasible, nor might it be desirable, to bring together all data in one place. For such constellation, a federated data environment is an appropriate alternative. This may be particularly beneficial in aggregating safety information from different trials, possibly enlarging the knowledge base on less frequent adverse events. Due to lack of time in the workshop agenda, there was no dedicated discussion group on how detection of long-term side effects could be improved as part of an APT approach.

In summary, statistical analysis tools both adequate and flexible must be made available to process complex multivariate and hierarchical data, also from various data sources. Not only frequentist and likelihood methodology can be used to this effect, but also Bayesian techniques, allowing to keep pace with increasing and evolving evidence, through Bayesian learning [29].

### Research questions for the interpandemic interval

There is consensus that a vaccine APT set up in the context of pandemic preparedness should run perpetually, i.e., “ever-warm” or “warm-base” in the interpandemic interval, and have the built-in ability to pivot to pandemic mode at shortest notice.

Outside a pandemic, relevant research questions are constantly fed into a vaccine APT which should result in an uninterrupted flow of new evidence. Research questions of public health relevance will primarily emanate from research gaps left by industry trials. Findings will ideally support informed decision-making on pressing public health issues, expressly so in absence of a pandemic. This represents an important return on investment apart from keeping the entire APT infrastructure up and running, and thus ready to respond whenever a pandemic strikes.

Interpandemic vaccine APT trial activity should center around pathogens that cause acute respiratory infection (ARI). Candidate pathogens include – but are not limited to – RSV, SARS-CoV-2 and seasonal influenza, but also avian influenza as a pathogen of particularly high pandemic potential.

Investigations will preferentially focus on vulnerable populations, underrepresented in industry trials, notably the extremes of age (infants, children, > 65 years, > 80 years), pregnant and lactating women, and the immunocompromised with distinct subgroups as per underlying condition and treatments, as well as otherwise migrant and socio-economically disadvantaged. This would not preclude enrolling healthy all-comers in phase 2 or phase 3 trials (or phase 2/3 with seamless transition to phase 3 after successful phase 2) on candidate vaccines, primary endpoints being immunogenicity or clinical endpoints respectively. Approved vaccines are to be investigated preferably in vulnerable populations.

Research on approved vaccines should target label extension/modification or any impactful practice change. This pertains to alternative administration schedules in terms of fractionation, dose, heterologous regimen, interchangeability, or by alternative routes like intradermal for antigen-sparing or intranasal of an unapproved formulation. Other approaches may encompass head-to-head comparison, co-administration (also fixed combinations) and booster vaccinations. Head-to-head-studies may target different formulations of the same vaccine or vaccines based on different technology platforms but for the same pathogen. As overarching topics, the decline of protection over time, correlates-of-protection and cross-protection (heterologous immunity) should be explored whenever possible, with particular focus on cell-mediated immunity, as a potential predictor of vaccine efficacy.

A concrete example to start with would be a phase 2 APT sub-study on an avian influenza vaccine originating from academia or industry. Such sub-study would be conducted in a few top-tier sites in one or two countries, followed by a safety-only phase 3 at many trial sites and in several countries.

In candidate vaccines there is also the option to run a phase 2 human challenge sub-study on qualifying pathogens at a dedicated facility affiliated to the APT network. Heterologous immunity may be another topic to be addressed in a human challenge vaccine trial.

APT investigations in the interpandemic interval need to utilize a wide spectrum of clinical research activities to keep up quality at the trial sites, in terms of enrolment capacity, standards of documentation, workup of biosamples and any related logistics. Prospective observational studies that address topics of a different nature and that require a different set of skills would not adequately prepare trial sites for a pandemic emergency.

### Vaccine immunology

Vaccine immunology encompasses a broad range of immunological parameters that are measured with a variety of methods. Pathogen-specific considerations can guide the selection of relevant immunological parameters. At the same time, upfront definition of a more generic portfolio of immunoassays is required to assess immunogenicity of vaccines against any yet unknown Disease X of pandemic potential. Therefore, vaccine immunology capacity – including but not limited to in-depth elucidation of cell-mediated immunity – needs to be tailor-made to the requirements of a vaccine APT and is, as such, indispensable for state-of-the-art pandemic preparedness. A network of dedicated vaccine immunology laboratories should undertake essential and relevant research in the interpandemic interval but switch ad-hoc to coordinated high-throughput analyses in a pandemic emergency. Such a laboratory network can be organized as a vaccine immunology group that is strongly involved in the design, conduct and analysis of a vaccine APT (Fig. 1). The research agenda of the vaccine immunology group should be aligned with all strategic objectives and priorities of the APT, including target pathogens and populations. Further objectives are the generation of key knowledge on CoP, assessment of mucosal immunity, and comparisons of vaccine technology platforms and vaccination regimens/schedules. Inclusion of immunological endpoints as CoP is required as an APT evaluates novel vaccine platforms in different populations over time, i.e., while immunity at the population level continuously evolves.

A vaccine immunology group should have the capacity to perform core assays on key immunological parameters of systemic and mucosal immunity that may ultimately serve as primary endpoints. Such assays must meet regulatory requirements. Continuous exchange with regulators on immunological endpoints and immunoassays is therefore crucial. Validation of prototype assays in the interpandemic interval accelerates the validation for pathogen-specific assays in a pandemic emergency. Today, antibody analyses are most amenable to such requirements, but cellular immune response may play a central role in assessing immunity against a specific Disease X. Hence, any effort should be undertaken in a vaccine APT to assess cell-mediated immunity. Vaccine immunogenicity must also be comprehensively evaluated including non-neutralizing functions of antibodies and cellular immune responses at mucosal surfaces. The vaccine immunology group within an APT needs to collaborate closely with other research laboratories outside the APT that are specialized in exploratory studies of human immune response. This allows for high dimensional analysis of cellular and molecular components increasing the value of information generated in the APT. A fundamental prerequisite for interoperability between laboratories is the creation of a vocabulary of immunology data, so far not adopted in Europe.

Another concern in pandemic emergency is the prompt and uninterrupted supply of high-quality reagents for immunoassays. During the interpandemic interval, efficient communication channels need to be established with initiatives that pursue complementary activities (in the EU and beyond) to promote sharing of methods and reagents as well as harmonization of relevant practices.

In summary, the success of a vaccine APT critically depends on expertise in vaccine immunology. Such expertise can best be leveraged through a vaccine immunology group as a core structure within an APT.

### A consortium focused on APT, before and during pandemic emergencies

**Fig. 2:**
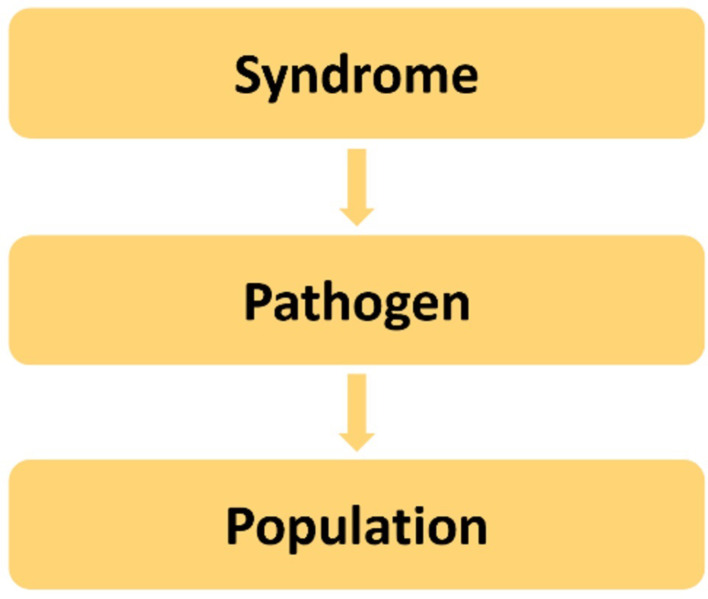
Structuring the overall approach of a vaccine APT

APT design is the methodology of choice for perpetually running vaccine trials in the pandemic preparedness arena. This reflects WHO requirements for the future global clinical trial ecosystem, pertaining also to optimal scientific and ethical design, excellence in statistical methods, development of clinical trial site capacity and enrolment into large clinical trials [1]. A consortium that is to conduct one or more vaccine APTs should structure its overall approach first by syndromes, then different pathogens and, subsequently, by populations (Fig. 2). Acute respiratory infection (ARI) pathogens of pandemic potential – with avian flu ranking top – should be the topic focus of a vaccine APT consortium both before and in pandemic emergency. Such portfolio may be extended to non-ARI pathogens of lower pandemic potential, such as enteroviruses (E71, polio), Ebola, Lassa, and vector-borne disease, e.g. West Nile virus. Candidate research questions for a vaccine APT must be validated outside the APT consortium in an unbiased manner. Therefore, a European vaccine APT consortium should be fully integrated into the decision-making matrix at the European level, with the European Commission’s Clinical Trial Coordination Mechanism (formerly: Coordinating Committee) [30] as prioritization body and CoMeCT [31] with its subpanels for scientific assessment and coordination (Fig. 1) as per current state of discussions, both in and outside emergencies. Such setup would also ensure formal communication channels between a vaccine APT consortium and the European Medicines Agency (EMA), the European Commission, the Health Emergency Response Authority (HERA), the European Centre for Disease Control (ECDC), and the European Pandemic Preparedness Partnership as developed by BE READY [32]. At the level of the APT consortium itself, decision-making and coordination are to be kept as lean and agile as possible with a Coordination Board, two adjunct advisory boards, the APT-specific panels referred to in the results section on Statistics and APT Methodology, and centres of competence with the Expert Panel of Vaccine Trialists and the Vaccine Immunology Group (Fig. 1).

**Fig. 3:**
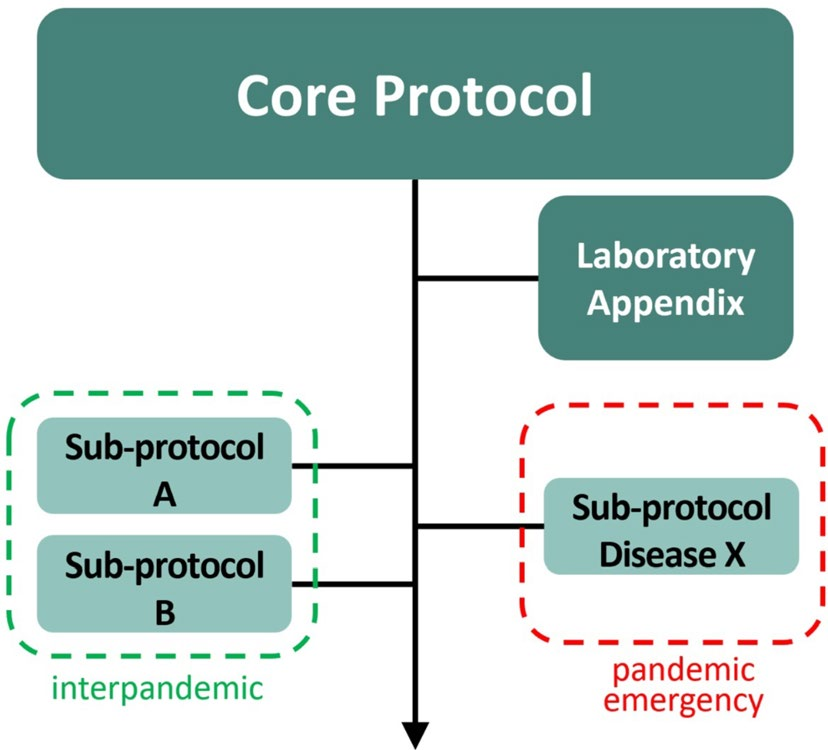
Prototypical APT protocol structure

For a visualization of a prototypical protocol structure see Fig. 3. An APT sub-study, both in and outside a pandemic may be conducted in phase 2-like settings with hundreds of vaccinees, assigned to experienced clinical trial sites with high volume enrolment capacity, preferably in only one country to control for administrative complexity and cost. Several phase 2 sub-studies may be conducted in parallel (including phase 2 human challenge vaccine trials), each in a different country, addressing different questions. For vaccines still under development for ultimate marketing authorization, an APT consortium may collaborate with vaccine developers from academia and industry. Substantial public funding, from the EU and its member states, would be needed in phase 2 and 3 for vaccines that originate from academia or small and medium-sized companies.

As a specific provision for the pandemic emergency, “template” Disease X sub-protocols for pathogens yet unknown must be developed and stockpiled by a vaccine APT consortium. These sub-protocols of syndrome-oriented designs cover ARI-settings and non-ARI-settings, for phase 2 and for phase 3 (or phase 2/3). Disease X sub-protocols are best updated every 2 years and submitted to regulators and ethics committees. This “dry-run” is aligned with table-top exercises of the European Pandemic Prepared Partnership for pressure-testing of emergency procedures. In the event of a pandemic emergency, the European Commission’s Clinical Trial Coordination Mechanism will prioritize vaccine trials, ensure commensurate funding and access to vaccine compound for execution of one or more of the Disease X sub-protocols in pandemic mode.

There are particular challenges of a vaccine APT as opposed to a treatment APT. E.g., for adaptations linked to a CoP, laboratory results need to be made available fast enough so that adaptations can be implemented while a substantial number of vaccinees have not been enrolled yet. This is of unique relevance given the relatively short enrolment periods of vaccine trials. Therefore, centralized capacity in highly specialized immunology laboratories for high-throughput analyses is a must. In contrast, laboratory capacity at the trial sites can be limited to basic serology for eligibility purposes. In a treatment trial, patients seek treatment and need to be convinced to join a trial. In the vaccine setting, volunteers need to be convinced to get vaccinated in the first place and then to join a trial on top. Hence, a volunteer registry, like the one of VACCELERATE [10], is an important tool to ensure speedy enrolment into a vaccine APT while increasing public awareness and fostering citizen engagement. Another example for accelerating trial conduct is the VACCELERATE site network [6], which facilitates access to trial sites that are differentiated by their experience and enrolment capacity (top-tier vs. lower-tier) specifically for vaccine trials. Complementing work areas in an APT consortium are FAIRification of data (also relevant for importing data to be used in disease modelling), research on statistical methodology, health communication & training directed at healthcare providers, patients and the public, Good Participatory Practice for Trials of (re-)Emerging Pathogens (GPP-EP as per WHO definition [33]), health economics, interaction with social sciences and humanities, and international outreach beyond the European Region.

A SWOT analysis identified several threats to a vaccine APT. In the European Union, CTIS (Clinical Trials Information System) provides the strength of one-stop shopping for clinical trial submission to both competent authorities and ethics [34]. However, CTIS is currently incompatible with the APT approach, the most salient issues being the lack of parallel processing of amendments and timelines. Besides, there is a continued lack of harmonization across Europe [30] regarding ethics requirements of informed consent forms, biobanking concepts and data protection. For a vaccine APT in pandemic preparedness the initial planning period should be at least 10 years, but no long-term funding instruments are available as of now.

## Discussion

There was consensus among the overwhelming majority of experts at the workshop that APT methodology can indeed be applied to vaccine trials. This is further supported by the existing blueprint of a large-scale vaccine APT developed for an outbreak of Marburg virus, with potentially tens of thousands of vaccinees [18]. Undoubtedly, APT designs are more complex but have the potential to accelerate knowledge generation [13]. There was agreement among workshop attendees that, regardless of design complexity, execution at the trial sites must be simple and straightforward. A prerequisite of a vaccine APT is the built-in ability to pivot to crisis mode, i.e., to promptly activate Disease X sub-studies, once a pandemic emergency is declared. Also, research questions fed into a vaccine APT in the interpandemic interval should address relevant public health needs, thereby ensuring a constant return on investment. This will garner societal support for commensurate and sustainable funding.

Particular importance was assigned to the value of soft factors in keeping a clinical research network alive, trust being of particular importance for complex adaptive endeavours [35]. Leadership in pandemic emergency and integrated pandemic response mechanisms being vital on top [36].

As one discussant put it: The future is not about whether or not to implement adaptive design features; the future is only about to what degree adaptive features will be implemented in a *state-of-the-art* trial. This will likely contribute to a paradigm shift from “traditional” RCTs to the concept of continuously learning adaptive platform trials [13].

## Data Availability

No datasets were generated or analysed during the current study.

## References

[1] Moorthy V, Abubakar I, Qadri F, Ogutu B, Zhang W, Reeder J, Farrar J. The future of the global clinical trial ecosystem: a vision from the first WHO Global clinical trials Forum. Lancet. 2024;403:124–6. 38128557 10.1016/S0140-6736(23)02798-8

[2] Heath PT, Preg-CoV -. A Phase II, randomised, single-blind, platform trial to assess safety, reactogenicity and immunogenicity of COVID-19 vaccines in pregnant women in the United Kingdom. 2022. https://vaccine.ac.uk/wp-content/uploads/2022/05/Preg-COV_Study-Protocol_v9.0_14.04.2022_clean.pdf (laccessed Feb 13 2024).

[3] McLeod C, Ramsay J, Flanagan KL, Plebanski M, Marshall H, Dymock M, et al. Core protocol for the adaptive platform trial in COVID-19 vaccine priming and BOOsting (PICOBOO). Trials. 2023;24:202. 36934272 10.1186/s13063-023-07225-z PMC10024280

[4] Written summary of an online meeting with representatives from the European Commission. Directorate-General Research & Innovation, and VACCELERATE, held on February 3rd, 2023. Document filed in the European Commission’s official document filing system ARES under the reference Ares(2023)881735. Document available upon request from the corresponding author of this article.

[5] Goossens H, Derde L, Horby P, Bonten M. The European clinical research response to optimise treatment of patients with COVID-19: lessons learned, future perspective, and recommendations. Lancet Infect Dis. 2022;22:e153–8. 34951954 10.1016/S1473-3099(21)00705-2 PMC8691848

[6] Salmanton-García J, Wipfler P, Valle-Simon P, Merakou C, Kopsidas I, Bethe U, et al. VACCELERATE Site Network: real-time definition of clinical study capacity in Europe. Vaccine. 2023;41:3915–22. 37210309 10.1016/j.vaccine.2023.05.006 PMC10194816

[7] Kenny G, O’Reilly S, Wrigley Kelly N, Negi R, Gaillard C, Alalwan D, et al. Distinct receptor binding domain IgG thresholds predict protective host immunity across SARS-CoV-2 variants and time. Nat Commun. 2023;14:7015. 37919289 10.1038/s41467-023-42717-1 PMC10622572

[8] Neuhann JM, Stemler J, Carcas A, Frias-Iniesta J, Bethe U, Heringer S, et al. A multinational, phase 2, randomised, adaptive protocol to evaluate immunogenicity and reactogenicity of different COVID-19 vaccines in adults >/=75 already vaccinated against SARS-CoV-2 (EU-COVAT-1-AGED): a trial conducted within the VACCELERATE network. Trials. 2022;23:865. 36209129 10.1186/s13063-022-06791-y PMC9547672

[9] Neuhann JM, Stemler J, Carcas AJ, Frias-Iniesta J, Akova M, Bethe U, et al. Immunogenicity and reactogenicity of a first booster with BNT162b2 or full-dose mRNA-1273: a randomised VACCELERATE trial in adults >/=75 years (EU-COVAT-1). Vaccine. 2023;41:7166–75. 37919141 10.1016/j.vaccine.2023.10.029

[10] Salmanton-García J, Stewart FA, Heringer S, Koniordou M, Alvarez-Barco E, Argyropoulos CD, et al. VACCELERATE Volunteer Registry: a European study participant database to facilitate clinical trial enrolment. Vaccine. 2022;40:4090–7. 35659449 10.1016/j.vaccine.2022.05.022 PMC9159788

[11] Koenig F, Spiertz C, Millar D, Rodriguez-Navarro S, Machin N, Van Dessel A, et al. Current state-of-the-art and gaps in platform trials: 10 things you should know, insights from EU-PEARL. EClinicalMedicine. 2024;67:102384. 38226342 10.1016/j.eclinm.2023.102384 PMC10788209

[12] Hofner B, Asikanius E, Jacquet W, Framke T, Oude Rengerink K, Aguirre Dávila L, et al. Vaccine Development during a pandemic: General lessons for clinical Trial Design. Stat Biopharm Res. 2023;0:1–13.

[13] The Adaptive Platform Trials Coalition. Adaptive platform trials: definition, design, conduct and reporting considerations. Nat Rev Drug Discov. 2019;18:797–807. 31462747 10.1038/s41573-019-0034-3

[14] Rothoeft T, Maier C, Talarico A, Hoffmann A, Schlegtendal A, Lange B et al. Natural and hybrid immunity after SARS-CoV-2 infection in children and adolescents. Infection. 2024. 10.1007/s15010-024-02225-w PMC11288991 38499828

[15] Bofill Roig M, Burgwinkel C, Garczarek U, Koenig F, Posch M, Nguyen Q, Hees K. On the use of non-concurrent controls in platform trials: a scoping review. Trials. 2023;24:408. 37322532 10.1186/s13063-023-07398-7 PMC10268466

[16] Liu M, Li Q, Lin J, Lin Y, Hoffman E. Innovative trial designs and analyses for vaccine clinical development. Contemp Clin Trials. 2021;100:106225. 33227451 10.1016/j.cct.2020.106225 PMC7834363

[17] Stallard N, Hampson L, Benda N, Brannath W, Burnett T, Friede T, et al. Efficient adaptive designs for clinical trials of interventions for COVID-19. Stat Biopharm Res. 2020;12:483–97. 34191981 10.1080/19466315.2020.1790415 PMC8011600

[18] Longini IM, Yang Y, Fleming TR, Munoz-Fontela C, Wang R, Ellenberg SS, et al. A platform trial design for preventive vaccines against Marburg virus and other emerging infectious disease threats. Clin Trials. 2022;19:647–54. 35866633 10.1177/17407745221110880 PMC9679315

[19] Meyer ELMT, Parke T, Jacko P, Franz Koenig F. SIMPLE - a modular tool for simulating complex platform trials. SoftwareX; 2023. 10.1016/j.softx.2023.101515 (accessed Mar 1 2024).

[20] Willem L, Verelst F, Bilcke J, Hens N, Beutels P. Lessons from a decade of individual-based models for infectious disease transmission: a systematic review (2006–2015). BMC Infect Dis. 2017;17:612. 28893198 10.1186/s12879-017-2699-8 PMC5594572

[21] Collignon O, Schiel A, Burman CF, Rufibach K, Posch M, Bretz F. Estimands and Complex innovative designs. Clin Pharmacol Ther. 2022;112:1183–90. 35253205 10.1002/cpt.2575 PMC9790227

[22] Michiels H, Vandebosch A, Vansteelandt S. Estimation and interpretation of vaccine efficacy in COVID-19 randomized clinical trials. Stat Commun Infect Dis. 2022;14.

[23] Alonso A, Bigirumurame T, Burzykowski T, Buyse M, Molenberghs G, Muchene L, et al. Applied Surrogate Endpoint evaluation with SAS and R. Boca Raton: Chapman & Hall/CRC; 2017.

[24] Collignon O, Burman CF, Posch M, Schiel A. Collaborative platform trials to fight COVID-19: Methodological and Regulatory considerations for a Better Societal Outcome. Clin Pharmacol Ther. 2021;110:311–20. 33506495 10.1002/cpt.2183 PMC8014457

[25] Collignon O, Gartner C, Haidich AB, James Hemmings R, Hofner B, Petavy F, et al. Current statistical considerations and Regulatory perspectives on the planning of Confirmatory Basket, Umbrella, and platform trials. Clin Pharmacol Ther. 2020;107:1059–67. 32017052 10.1002/cpt.1804

[26] CHMP. Concept paper on platform trials. Amsterdam: European Medicines Agency; 2022.

[27] Chalupka A, Richter L, Chakeri A, El-Khatib Z, Theiler-Schwetz V, Trummer C et al. Effectiveness of a fourth SARS-CoV-2 vaccine dose in previously infected individuals from Austria. Eur J Clin Invest. 2023:e14136. 10.1111/eci.14136 PMC11475503 38032853

[28] Hattab D, Amer MFA, Al-Alami ZM, Bakhtiar A. SARS-CoV-2 journey: from alpha variant to omicron and its sub-variants. Infection. 2024;52:767–86. 38554253 10.1007/s15010-024-02223-y PMC11143066

[29] Breen R. Beliefs, rational choice and bayesian learning. Rationality Soc. 1999;11:463–79.

[30] EMA. Report of the EMA/ETF workshop on Lessons Learned on Clinical Trials in Public Health Emergencies. 2023. https://www.ema.europa.eu/en/documents/report/report-emaetf-workshop-lessons-learned-clinical-trials-public-health-emergencies_en.pdf (accessed Mar 4 2024).

[31] Coordination Mechanism for Cohorts and Clinical Trials. (CoMeCT). https://comectproject.org/ (accessed Mar 4 2024).

[32] BE READY Coordination and Support Action. Building a European Partnership for Pandemic Preparedness.

[33] WHO. Good participatory practice guidelines for trials of emerging (and re-emerging) pathogens that are likely to cause severe outbreaks in the near future and for which few or no medical countermeasures exist (GPP-EP). Outcome document of the consultative process. Geneva: Word Health Organization; 2016. https://cdn.who.int/media/docs/default-source/blue-print/good-participatory-practice-for-trials-of-(re-)emerging-pathogens-(gpp-ep)_guidelines.pdf?sfvrsn=102e3e. 70_9&download=true (accessed Mar 5 2024).

[34] European Medicines Agency. Clinical Trials Information System. https://www.ema.europa.eu/en/human-regulatory-overview/research-and-development/clinical-trials-human-medicines/clinical-trials-information-system (accessed Mar 5 2024).

[35] WHO. Ethics and adaptive platform trial design in public health emergencies: meeting report. Geneva, Switzerland: World Health Organization. 2023. https://www.who.int/publications/i/item/9789240079670 (accessed Feb 19 2024).

[36] Goldschmidt PG. At the end of every pandemic: beginning a pandemic playbook to Respond to the Next. Front Public Health. 2022;10:838561. 35570978 10.3389/fpubh.2022.838561 PMC9093215

[37] Dimairo M, Pallmann P, Wason J, Todd S, Jaki T, Julious SA, et al. The adaptive designs CONSORT Extension (ACE) statement: a checklist with explanation and elaboration guideline for reporting randomised trials that use an adaptive design. BMJ. 2020;369:m115. 32554564 10.1136/bmj.m115 PMC7298567

[38] Roig MB, Krotka P, Burman CF, Glimm E, Gold SM, Hees K, et al. On model-based time trend adjustments in platform trials with non-concurrent controls. BMC Med Res Methodol. 2022;22:228. 35971069 10.1186/s12874-022-01683-w PMC9380382

[39] Saville BR, Berry DA, Berry NS, Viele K, Berry SM. The bayesian time machine: accounting for temporal drift in multi-arm platform trials. Clin Trials. 2022;19:490–501. 35993547 10.1177/17407745221112013

